# Hochuekkito Combined with Pulmonary Rehabilitation in Apathetic Patients with Chronic Obstructive Pulmonary Disease: A Randomized Controlled Pilot Trial

**DOI:** 10.3390/jcm11195673

**Published:** 2022-09-26

**Authors:** Hironobu Hamada, Kiyokazu Sekikawa, Ken Okusaki, Takefumi Dodo, Kazuyoshi Kagawa, Tatsuya Sumigawa, Yoshikazu Awaya, Naoki Sakimoto, Sachiko Shioya, Keisuke Hakozaki, Toru Kadowaki, Maki Kakimoto, Ryoji Ito, Koji Kawamichi, Keiichi Kondo, Haruchi Namba, Hiroshi Iwamoto, Noboru Hattori

**Affiliations:** 1 Department of Physical Analysis and Therapeutic Sciences, Graduate School of Biomedical and Health Sciences, Hiroshima University, Hiroshima 734-8551, Japan; 2 Department of Molecular and Internal Medicine, Graduate School of Biomedical and Health Sciences, Hiroshima University, Hiroshima 734-8551, Japan; 3 Department of Internal Medicine, Mihara Medical Associations Hospital, Mihara 723-0051, Japan; 4 Department of Respiratory Medicine, Hiroshima City Medical Association-Administered Hiroshima City Aki Hospital, Hiroshima 736-0088, Japan; 5 Department of Respiratory Medicine, Miyoshi Central Hospital, Miyoshi 728-8502, Japan; 6 Department of Respiratory Medicine, Innoshima Medical Association Hospital, Onomichi 722-2211, Japan; 7 Department of Pulmonary Medicine, National Hospital Organization Matsue Medical Center, Matsue 690-8556, Japan; 8 Department of Rehabilitation, National Hospital Organization Matsue Medical Center, Matsue 690-8556, Japan; 9 Department of Respiratory Medicine, National Hospital Organization Ehime Medical Center, Toon 791-0281, Japan; 10 Department of Rehabilitation, National Hospital Organization Ehime Medical Center, Toon 791-0281, Japan; 11 Department of Respiratory Medicine, Tadanoumi Hospital, Takehara 729-2316, Japan

**Keywords:** chronic obstructive pulmonary disease, apathy, dyspnea, health-related quality of life, physical activity, Hochuekkito

## Abstract

The main treatment goals for chronic obstructive pulmonary disease (COPD) are the reduction of its symptoms and future risks. The addition of the traditional herbal medicine Hochuekkito (TJ-41) treatment to pulmonary rehabilitation (PR) has been reported to improve dyspnea and health-related quality of life (HRQOL) in patients with COPD. However, the reason for this improvement is not sufficiently understood. The purpose of the present study was to investigate whether the addition of TJ-41 treatment to PR improves symptoms of apathy, dyspnea, and HRQOL and increases physical activity among apathetic patients with COPD. Apathetic patients with COPD were randomly assigned to receive low-intensity exercise with (TJ-41 group) or without (control group) TJ-41 treatment for 12 weeks. A total of 29.9% of COPD patients had apathetic symptoms without severe depression. After the 12-week treatment, Apathy Scale, Patient Health Questionnaire-9, visual analog scale for dyspnea, and COPD assessment test energy scores decreased significantly in the TJ-41 group (*p* < 0.05), but not in the control group. Additionally, the total number of steps taken was significantly higher in the TJ-41 group than in the control group. TJ-41 combined with PR may benefit apathetic patients with COPD with respect to apathy, dyspnea, HRQOL, and physical activity, but larger randomized placebo-controlled trials are required to validate the findings because of the small sample size and lack of placebo controls in this study.

## 1. Introduction

Chronic obstructive pulmonary disease (COPD) is a lung disease characterized by persistent respiratory symptoms and airflow limitation due to airway and alveolar abnormalities caused by long-term inhalation of toxic substances, such as tobacco smoke, and is influenced by host factors, including abnormal lung development [1].

The main treatment goals for COPD include reduction of symptoms and future risks [1]. Symptom relief, improvement of health-related quality of life (HRQOL), and exercise tolerance lead to reduced symptoms [1]. In addition, improving physical activity as well as exercise tolerance is considered to be important in the management of COPD because physical activity level is associated with mortality, acute exacerbations, hospitalization, and HRQOL [1,2,3]. Pulmonary rehabilitation (PR) is a useful non-pharmacological therapy that improves dyspnea, HRQOL, and exercise tolerance in patients with COPD [1]. We hypothesized that some Japanese herbal medicines might have a synergistic effect with PR to improve symptoms in patients with COPD because they had been used to improve the physical and mental conditions of patients suffering from various diseases.

Hochuekkito (TJ-41) is used to treat patients with chronic diseases and post-illness weakness. We previously conducted a clinical study to evaluate the efficacy and safety of adding TJ-41 treatment to PR in malnourished patients with COPD. Our previous study demonstrated that the addition of TJ-41 treatment to PR improved body weight, dyspnea scores, and HRQOL scores in patients with COPD [4]. However, the reason for the improvement in dyspnea and HRQOL scores after TJ-41 administration was unclear.

Several reports have demonstrated that many patients with COPD have clinically significant levels of depression [5,6] and that patients with COPD and depression had a significantly increased dyspnea and decreased HRQOL compared to those without depression [6,7]. Previous evidence indicates that TJ-41 has an anti-depressive effect in a mouse model of depression with learned helplessness behavior [8]. Apathy (a lack of motivation) is a common neuropsychiatric symptom that complicates many age-related disorders including Alzheimer’s disease, Parkinson’s disease, and stroke, and is distinct from depression [9,10,11]. Previous reports suggest that apathy has been observed in the elderly with chronic diseases [12,13,14,15], although, there has been no report to show the prevalence of apathetic patients with COPD. Apathy, rather than depression, may be more closely related to the role of TJ-41 in dyspnea and HRQOL improvement in our previous study because TJ-41 has been used to increase motivation [16], and has shown to improve motivation in patients with COPD [17]. In addition, increased motivation may be an influencing factor for improvement in exercise tolerance in patients with COPD undergoing PR [18]. Apathy is a predictor of poor functional recovery after stroke during rehabilitation [11,19]. Furthermore, it is negatively associated with physical activity among the elderly [20].

We hypothesized that the addition of TJ-41 treatment to PR would improve apathy, dyspnea, and HRQOL, leading to increased physical activity in apathetic patients with COPD. In this study, we investigated the efficacy and safety of TJ-41 in apathetic patients with COPD who underwent PR.

## 2. Materials and Methods

### 2.1. Study Design

This 12-week, open-label, randomized, parallel-group comparative pilot study of TJ-41 administration during PR was conducted at eight clinical hospitals, namely, Hiroshima University Hospital, Hiroshima, Japan, Mihara Medical Association Hospital, Mihara, Japan, Hiroshima City Medical Association-administered Hiroshima City Aki Hospital, Hiroshima, Japan, Miyoshi Central Hospital, Miyoshi, Japan, and Tadanoumi Hospital, Takehara, Japan, Innoshima Medical Association Hospital, Onomichi, Japan, Matsue Medical Center, Matsue, Japan, and Ehime Medical Center, Toon, Japan between April 2018 and March 2020. The study was approved by the institutional ethics committee of each hospital and further approved by the Hiroshima University Certified Review Board (more information in the Institutional Review Board Statement section). Written informed consent was obtained from all patients. This study was registered in the University Hospital Medical Information Network of Japan (http://www.umin.ac.jp/ctr/ (accessed on 14 August 2022); registration number, UMIN000029368) and the Japan Registry of Clinical Trials (https://jrct.niph.go.jp/ (accessed on 14 August 2022); registration number, jRCTs061180042).

### 2.2. Patients

The inclusion criteria were as follows: (i) moderate to severe COPD, defined by forced expiratory volume in 1 s/forced vital capacity (FEV1/FVC) < 70% and FEV1% predicted to be >30% and <80%, that is, stages II and III, based on Global Initiative for Chronic Obstructive Lung Disease (GOLD) classification [1]; (ii) low levels of motivation, i.e., an Apathy Scale (AS) score ≥ 16 points [21,22]; (iii) clinically stable disease and ability to participate in PR for 12 weeks; (iv) age ≥ 40 years; and (v) smoking history of 10 pack-years or more.

Patients with the following characteristics were excluded from the study: (i) with severe mental illness, i.e., a Patient Health Questionnaire-9 (PHQ-9) score ≥ 15 points [23]; (ii) received PR within 24 weeks preceding the study; (iii) diagnosed with other pulmonary diseases or alpha1-antitrypsin deficiency; (iv) diagnosed with an acute exacerbation within 4 weeks prior to the start of the study; (v) received pulmonary transplantation; (vi) received herbal medicine for any problem within 4 weeks preceding the study; (vii) had newly received bronchodilators, or inhaled or systemic corticosteroids within 2 weeks before the study; (viii) diagnosed with other severe diseases, such as malignant tumors, and autoimmune liver, renal, heart, hematologic, or metabolic disease; (ix) participated in another clinical trial within 4 weeks preceding the study; (x) definitely or possibly pregnant; or (xi) judged by the physician as inappropriate for participation in the present study.

This was a prospective pilot study. The sample size was calculated based on the number of apathetic patients with COPD undergoing PR per year at the research institutions, which was set as the number of cases considered to be feasible in daily medical practice within the registration period.

### 2.3. Study Protocol

Randomization was performed manually using a central registration system. Patients who met the eligibility criteria were enrolled and randomly assigned to receive PR with Group A, which include patients who were treated with TJ-41 powder (2.5 g TJ-41, Extract Granules for Ethical Use, Tsumura Co., Tokyo, Japan), administered orally (2.5 g) three times per day before each meal or between meals, or Group B, which included patients who were not treated with TJ-41 powder. Patients who met the enrollment criteria were randomized in a 1:1 ratio and stratified according to the clinical stage of COPD. Within 4 weeks of enrollment and randomization, the baseline number of daily steps was counted using a pedometer (H-236, DRETEC Co., Ltd., Saitama, Japan) for at least 2 weeks. Hospital visits were scheduled on the first day of administration of the study drug and PR. The number of daily steps and PR progress were regularly confirmed every 2 weeks throughout the 12-week treatment period.

Patients were permitted to use procaterol and sarbutamol metered-dose inhalers when needed, as well as a fixed dose of theophylline, long-acting β2-agonists and muscarinic antagonists, or inhaled and oral corticosteroids throughout the study period. Any additional medications, such as antibiotics and systemic corticosteroids, were permitted to control acute exacerbations.

PR was performed according to previously established methodology [4].

### 2.4. Test Drug

We used TSUMURA Hochuekkito extract granules for ethical use (TJ-41, Tsumura & Co., Tokyo, Japan; refer to http://mpdb.nibiohn.go.jp/stork/ (accessed on 14 August 2022)), which comprises 10 herbal medicines, as previously reported [4].

### 2.5. Outcome Measurements

The primary outcomes were changes in AS scores and the total number of steps. The secondary outcomes were changes in body weight, body mass index (BMI), pulmonary function, PHQ-9 score, modified Medical Research Council (mMRC) dyspnea scale score, visual analog scale (VAS) score for dyspnea, VAS score for fatigue, COPD assessment test (CAT) score, number of acute exacerbations, and completion rate of PR.

Lack of motivation was assessed using the Japanese version of the AS modified by Starkstein [21]. The AS score measures apathy symptoms based on 14 questions ranging from 0 (not at all) to 3 (a lot), with higher scores indicating more severe apathy symptoms. Based on a study of apathy in Japanese patients with stroke [22], an AS score ≥ 16 was regarded as a decrease in motivation. The number of daily steps measured by a pedometer was recorded in a diary and checked by respiratory therapists every two weeks. Depressive symptoms were determined using the PHQ-9, which is a well-validated, Diagnostic and Statistical Manual of Mental Disorders-Fourth Edition (DSM-IV) criterion-based measure for diagnosing depression and consists of 9 items ranging from 0 (not at all) to 3 (nearly every day). A PHQ-9 score ≥ 15 was considered indicative of severe depressive symptoms [23].

Body weight, BMI, pulmonary function, and mMRC dyspnea scale, VAS for dyspnea, VAS for fatigue, and CAT score were measured as previously described [4]. The criteria for acute exacerbations were in accordance with those used in our previous study [4].

Pulmonary function tests, and mMRC dyspnea scale, total CAT, and PHQ-9 scores were measured at baseline and after 12 weeks. Body composition parameters and AS, VAS for dyspnea, and VAS for fatigue scores were measured every four weeks. The number of steps taken was measured daily throughout the study period.

### 2.6. Safety Measurements

Adverse events were assessed throughout the 12-week study period in accordance with our previous study [4].

### 2.7. Statistical Analyses

Results are presented as mean ± standard deviation. The average number of daily steps taken two weeks prior to each visit was used in the analysis. The ratio of the number of steps after 4, 8, and 12 weeks of treatment to the baseline number of steps (i.e., the ratio of the number of steps) was used because the number of steps differed for each patient. The time-effect curve (area under the curve: AUC) was determined by multiplying the ratio of the number of steps with the time interval according to the trapezoidal rule. The AUC for the entire treatment period was calculated and defined as the total number of steps in each group. Partially missing data from the clinical evaluation were carried forward using the principle of last observation. The chi-squared (χ^2^) and Wilcoxon rank-sum test were used to compare data between the groups. The Wilcoxon signed-rank test was used to compare data between baseline and weeks 4, 8, and 12. All statistical analyses were performed using SPSS version 27 (IBM Japan Ltd., Tokyo, Japan). Statistical significance was set at *p* < 0.05.

## 3. Results

### 3.1. Patients’ Characteristics

**Figure 1:**
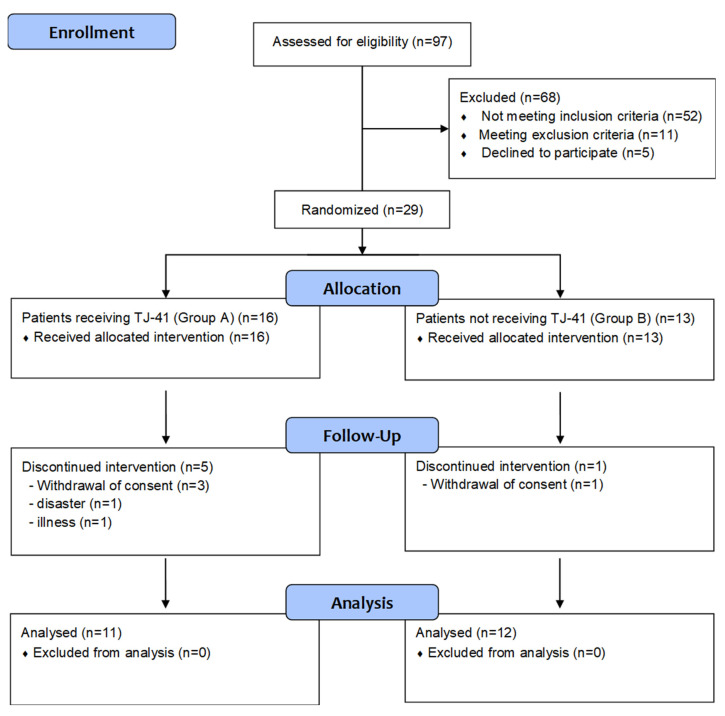
CONSORT flow diagram.

A flowchart displaying the study procedures is shown in Figure 1. A total of 97 patients were assessed for eligibility, of which 68 patients who did not meet the inclusion criteria, but met the exclusion criteria, or declined to participate were excluded. Twenty-nine patients with COPD had apathetic symptoms without moderate to severe depression and were randomized; five patients in group A and one patient in group B were excluded due to consent withdrawal, disaster, or illness. Therefore, 23 patients were enrolled in this study. Eleven patients (5 at stage II and 6 at stage III) received TJ-41 (Group A), while 12 patients (5 at stage II and 7 at stage III) did not receive TJ-41 (Group B).

There were no significant differences in age, body weight, BMI, AS and PHQ-9 score, number of daily steps taken, or medical treatment between the groups at baseline (Table 1). In addition, no significant differences were observed in predicted FEV1%, and mMRC dyspnea scale, VAS for dyspnea, VAS for fatigue, or total CAT score between groups at baseline (Table 2).

### 3.2. Outcomes

**Figure 2:**
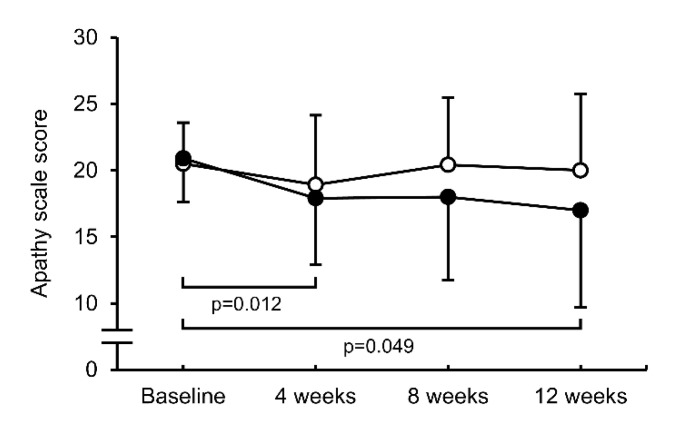
Changes in apathy scale score after 4, 8, and 12 weeks of treatment. Data are expressed as mean ± standard deviation. Closed circle: Group A, open circle: group B.

The AS score in group A significantly decreased after 4 and 12 weeks of treatment (*p* = 0.012 and *p* = 0.049, respectively) and tended to decrease after 8 weeks (*p* = 0.05), but no significant changes were observed in group B (Figure 2).

**Figure 3:**
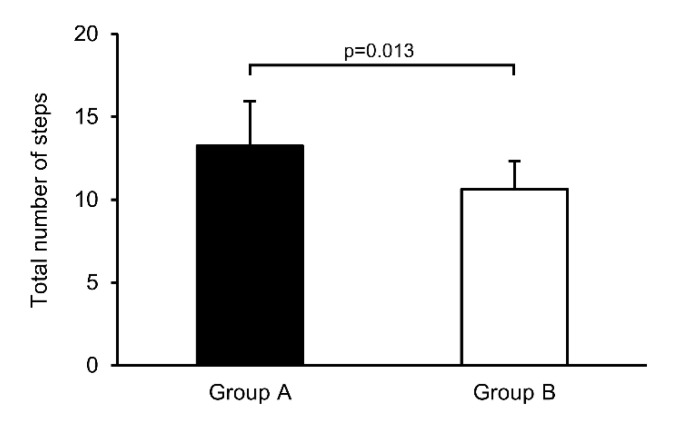
Total number of steps in both groups. Data are expressed as mean ± standard deviation.

The total number of steps taken in group A was significantly higher than that in group B (*p* = 0.013) (Figure 3).

The PHQ-9, VAS for dyspnea, and CAT score of energy in group A significantly decreased after 12 weeks of treatment (*p* = 0.031, *p* = 0.021 and *p* = 0.026, respectively), but no significant changes were observed in group B (Table 2).

The VAS for fatigue and CAT score of activity limitation in group B significantly decreased after the 12-week treatment (*p* = 0.023 and *p* = 0.005, respectively), but no significant changes were observed in group A (Table 2).

### 3.3. Adverse Events

One patient showed mild liver dysfunction and one had lumbar spinal canal stenosis. The causal relationship between these adverse events and TJ-41 remains unknown.

## 4. Discussion

Our study indicates that 29.9% of COPD patients have apathetic symptoms without severe depression. Adding TJ-41 treatment to PR significantly decreased AS, PHQ-9, VAS for dyspnea, and CAT energy scores. Additionally, the total number of steps taken was significantly higher in TJ-41-treated patients than in non-TJ-41-treated patients. TJ-41 combined with PR may benefit apathetic patients with COPD with respect to motivation, dyspnea, HRQOL, and physical activity. However, the limitations of this study (discussed below) such as the small sample size and open label method might have affected the validity of these findings, which would need to be addressed in the design of future trials.

To our knowledge, this is the first report to show the prevalence of apathy in patients with COPD. Chronic diseases such as cardiovascular diseases and diabetes mellitus (DM) have been reported to complicate apathy in patients [12,13,14,15]. The prevalence of apathy has been reported to be 29% in patients with cardiovascular diseases [12] and from 13.9% to 61.7% in patients with DM [14,15]. DM patients with apathy had higher BMI and were less likely to adhere to exercise plan compared to non-apathetic patients or administer the correct doses of insulin compared to non-apathetic patients [14]. The present study showed that 29.9% (29 of 97) of COPD patients had apathetic symptoms without moderate to severe depression. A larger clinical study is needed to clarify the prevalence of apathy in patients with COPD and characteristics of apathetic patients with COPD.

The addition of TJ-41 treatment to PR may improve apathy symptoms in patients with COPD. Tatsumi reported that administration of TJ-41 improved the motivation of patients [17]. Additionally, TJ-41 administration had shown improved CAT scores for energy in our previous study [4]. In the present study, the AS and CAT scores for energy significantly decreased in Group A, but not in Group B. The mechanism underlying the improvement in AS scores after TJ-41 administration for patients with COPD remains unknown. Some drugs that stimulate the acetylcholine or dopamine nervous system have been reported to improve apathetic symptoms [11,12,24]. Some components of TJ-41 may act on these nervous systems, although there are no reports of TJ-41 activating dopamine or acetylcholine in the nervous system. Inflammatory cytokines have also been reported to decrease dopaminergic function [25]. The anti-inflammatory effect of TJ-41 may improve dopaminergic function [26,27]. Further studies are needed to clarify the mechanism by which TJ-41 improves apathy symptoms in patients with COPD.

Improvement of apathy symptoms might improve dyspnea and HRQOL and increase physical activity in patients with COPD. Apathy interferes with the improvement of physical function in patients with stroke through rehabilitation [19] and is negatively associated with physical activity in older adults [20]. In the present study, TJ-41 administration significantly decreased AS, PHQ-9, VAS for dyspnea, and CAT energy scores in group A, while no change was observed in group B. Furthermore, the total number of steps taken was significantly higher in group A than in group B. Previous evidence indicates that a decrease in physical activity in patients with COPD leads to increased mortality, acute exacerbations, hospitalization, and decreased HRQOL [1,2,3]. No therapy to improve physical activity has been established, although internet- and pedometer-mediated interventions may be effective in improving physical activity [1,28,29]. The effects of bronchodilators or PR on physical activity have not yielded consistent results [28,30,31,32,33]. TJ-41 treatment combined with PR may be an important therapeutic strategy for treating patients with COPD. Larger clinical studies are needed to clarify the effects of TJ-41 on dyspnea, HRQOL, and physical activity in apathetic patients with COPD.

With regard to depression, COPD patients with depression presented higher dyspnea levels, and symptoms of depression were associated with a reduction in physical activity in patients with COPD [34,35]. Additionally, TJ-41 has been reported to improve depressive symptoms in experimental animal studies [8]. Comorbidities of apathy and depressive symptoms coexist in patients, post-stroke [11,36]. Therefore, we defined a cut-off value of 15 points on the PHQ-9 score to exclude severe depression cases from this study. As a result, only patients who represented mild depression on the PHQ-9 scores (4.1 ± 2.2 in group A and 4.2 ± 2.4 in group B) were enrolled. The improvement in apathy by TJ-41 treatment mainly contributed to improving dyspnea and increasing physical activity in this study.

The VAS for fatigue and CAT scores of activity limitation significantly improved in group B, but not in group A. This may be because the baseline VAS scores for fatigue and the baseline CAT scores of activity limitation were lower in group A than in group B, suggesting that there were fewer patients suffering from fatigue and activity limitation in group A. In particular, there were five patients with baseline CAT scores of 0 in group A, but only one in group B. Further, three patients receiving home oxygen therapy were included in group B but there were none in group A.

The exact frequency of adverse events after TJ-41 treatment is unknown because no studies have been conducted to determine the incidence of adverse events after TJ-41 treatment. However, previous reports have suggested a low incidence of significant adverse events such as interstitial pneumonia, pseudoaldosteronism, myopathy, liver dysfunction, and jaundice after TJ-41 treatment [37,38]. Furthermore, there have been no reports of lumbar spinal canal stenosis as an adverse event after TJ-41 treatment.

This study has several limitations. First, the number of patients was small. We screened approximately one hundred patients with COPD, but many patients were excluded due to various reasons such as the inclusion and exclusion criteria, refusal to participate, and withdrawal of previously obtained consent. Second, we did not use a placebo in our study. Creating a placebo for Japanese herbal medicines is challenging because of the distinctive bitter taste and flavor of these medicines. Sucrose and lactose, which are usually used as placebos, differ from Japanese herbal medicines. The beneficial outcomes of the TJ-41 treatment in combination with PR might have been overstated because of the small sample size and open label method of this study. On the other hand, the effects of the additional treatment, rather than TJ-41, on the outcome were thought to be insignificant because no patients with COPD needed any changes to their medications or PR, or were administered any additional medications due to acute exacerbations during the study period. Larger randomized placebo-controlled trials are required to validate the findings of this study.

## 5. Conclusions

Our results suggest that the addition of TJ-41 treatment to PR improves symptoms of apathy, dyspnea, and HRQOL and increases physical activity in apathetic patients with COPD. No adverse events with a causal relationship to TJ-41 were observed. TJ-41 treatment combined with PR may be a new therapeutic strategy for treating apathetic patients with COPD. However, the small sample size and open label method of this study might have affected the validity of these findings, which should be confirmed in future studies.

## Figures and Tables

**Table 1:** Baseline characteristics of patients with chronic obstructive pulmonary disease.

Variables	Group A (n = 11)	Group B (n = 12)	*p*-Value
Age (years)	72.5 ± 5.7	76.4 ± 7.1	0.169
Sex			
Male	10 (90.9)	11 (91.7)	0.949
Female	1 (9.1)	1 (8.3)	
Height (cm)	161.1 ± 4.6	164.4 ± 6.4	0.413
Body weight (kg)	53.3 ± 10.3	61.1 ± 7.9	0.151
Body mass index	20.5 ± 3.2	22.9 ± 2.7	0.069
GOLD stage			
Stage II	5 (45.5)	5 (41.7)	1.000
Stage III	6 (54.5)	7 (58.3)	
Apathy Scale score	20.9 ± 3.3	20.5 ± 3.1	0.833
PHQ-9	4.1 ± 2.2	4.2 ± 2.4	0.976
Number of daily steps	5056.5 ± 3929.2	3368.2 ± 1651.6	0.606
Medical treatment			
Use of LABA	6 (54.5)	9 (75.0)	0.304
Use of LAMA	10 (90.9)	12 (100.0)	0.286
Use of ICS	1 (9.1)	3 (25.0)	0.315
Home oxygen therapy	0 (0)	3 (25.0)	0.075

Data are expressed as the number (percentage) for categorical variables and mean ± standard deviation for numerical variables. Abbreviations: GOLD, Global Initiative for Chronic Obstructive Lung Disease; ICS, inhaled corticosteroid; LABA, long-acting β2-agonist; LAMA, long-acting muscarinic antagonist; PHQ-9, Patient Health Questionnaire-9.

**Table 2:** Changes in body composition, clinical symptoms, and HRQOL.

Variable	Group A (n = 11)			Group B (n = 12)		
	Baseline	12 Weeks	*p*-Value	Baseline	12 Weeks	*p*-Value
Body weight (kg)	53.3 ± 10.3	52.9 ± 11.1	0.484	61.1 ± 7.9	60.5 ± 7.2	0.230
Body mass index	20.5 ± 3.2	20.3 ± 3.6	0.594	22.9 ± 2.7	22.7 ± 2.4	0.508
Ideal body weight (% predicted)	92.9 ± 14.6	92.1 ± 16.2	0.594	103.1 ± 12.0	102.3 ± 10.6	0.563
Fat free mass (kg)	40.6 ± 5.8	40.5 ± 6.1	0.878	44.2 ± 6.4	43.9 ± 6.1	0.638
FVC (L)	2.8 ± 0.7	2.9 ± 0.6	0.415	2.9 ± 0.7	2.8 ± 0.8	0.328
FEV1 (L)	1.4 ± 0.4	1.4 ± 0.3	0.221	1.3 ± 0.4	1.3 ± 0.5	0.473
FEV1 (% predicted)	57.7 ± 16.2	58.1 ± 13.0	0.657	51.6 ± 18.6	51.8 ± 19.3	0.929
FEV1/FVC (%)	50.8 ± 8.6	49.5 ± 8.5	0.374	45.7 ± 10.9	47.1 ± 10.1	0.209
Apathy Scale score	20.9 ± 3.3	17.0 ± 7.3	0.049 *	20.5 ± 3.1	20.0 ± 5.8	0.959
PHQ-9	4.1 ± 2.2	2.5 ± 1.6	0.031 *	4.2 ± 2.4	2.9 ± 2.2	0.050
mMRC dyspnea scale score	1.4 ± 0.9	1.6 ± 0.9	0.480	1.6 ± 0.6	1.8 ± 0.8	0.317
VAS score for dyspnea	5.6 ± 2.9	2.4 ± 2.2	0.021 *	4.4 ± 1.6	3.5 ± 2.0	0.071
VAS score for fatigue	3.9 ± 2.3	2.1 ± 2.1	0.058	4.5 ± 2.4	2.5 ± 2.0	0.023 *
Total CAT score	13.8 ± 7.0	10.6 ± 6.2	0.220	15.6 ± 4.7	12.8 ± 3.5	0.152
Score of cough	1.2 ± 1.1	1.1 ± 0.8	0.792	1.8 ± 0.7	1.6 ± 1.7	0.729
Score of production of phlegm	1.7 ± 1.4	1.3 ± 1.2	0.194	1.4 ± 1.1	1.4 ± 1.0	1.000
Score of chest tightness	2.0 ± 0.9	1.7 ± 1.0	0.380	2.3 ± 1.2	2.0 ± 1.0	0.395
Score of breathlessness	3.5 ± 1.6	2.8 ± 1.7	0.167	3.7 ± 1.0	3.8 ± 0.9	0.480
Score of activity limitation	0.9 ± 1.1	0.7 ± 0.8	0.577	1.4 ± 0.9	0.5 ± 0.9	0.005 *
Score of confidence	1.0 ± 1.4	0.9 ± 1.2	0.914	1.7 ± 1.3	1.1 ± 1.3	0.160
Score of sleep	1.2 ± 1.3	0.9 ± 1.4	0.732	1.3 ± 1.2	0.8 ± 0.9	0.132
Score of energy	2.3 ± 0.9	1.2 ± 1.1	0.026 *	2.1 ± 1.0	1.6 ± 1.1	0.296

Data are expressed as mean ± standard deviation. * *p* < 0.05 vs. baseline. Abbreviations: CAT, chronic obstructive pulmonary disease assessment tests; FVC, forced vital capacity; FEV1, forced expiratory volume in 1 s; HRQOL, health-related quality of life; mMRC, modified Medical Research Council; PHQ-9, Patient Health Questionnaire-9; VAS, visual analog scale.

## Data Availability

All data generated or analyzed during this study are included in this published article.
